# Association among Olfactory Function, Lifestyle and BMI in Female and Male Elderly Subjects: A Cross-Sectional Study

**DOI:** 10.3390/nu15112492

**Published:** 2023-05-26

**Authors:** Giorgia Sollai, Roberto Crnjar

**Affiliations:** Department of Biomedical Sciences, University of Cagliari, 09042 Monserrato, Italy; crnjar@unica.it

**Keywords:** elderly subjects, physical activities, BMI, body weight, lifestyle, olfactory function, Sniffin’ Sticks

## Abstract

Physical activities seem to counteract the age-related physiological decline of the olfactory function which, influencing the food choices and eating behavior, can affect the body weight of individuals. The main purpose of this cross-sectional study was to evaluate the relationships between olfactory function and BMI in female and male Elderly Subjects (ES), according to the level of their lifestyle activities in physical, cognitive, and social terms. Considering weekly physical activities, the adult elderlies who decided to participate in this study were divided into active ES (*n* = 65) and non-active ES (*n* = 68). Assessment of weekly activities and olfactory function were performed by means of face-to-face interviews and the “Sniffin’ Sticks” battery test, respectively. The results show that ES who are overweight and with a non-active lifestyle achieved lower TDI olfactory scores than normal weight ES and those classified as active. Hyposmic and non-active ES showed a higher BMI than normosmic and active ES. Sex-related differences, with females performing better than males, were evident in the presence of at least one of the following conditions: non-activity, hyposmia, or overweight. Inverse correlations were found between BMI and TDI olfactory score and between BMI and hours/week spent on physical activities, both when subjects were considered all together and when they were divided into females and males. These findings suggest that a higher BMI is related to the olfactory dysfunction linked to active or non-active lifestyle and the sex-related differences, and the condition of hyposmia is related to the increase in body weight due to lifestyle and sex differences. Given that the relationship between BMI and non-exercise physical activities is comparable to that between BMI and exercise physical activities, and this may be of particular importance for ES with limited mobility.

## 1. Introduction

The human perception of both complex and simple odors is characterized by a great variability between individuals, due to the effect of multiple factors such as physiological, genetic, and environmental ones [1,2,3,4,5,6,7,8,9,10,11]. It is known that one of the main factors capable of negatively influencing the olfactory function is the natural aging process [12,13,14,15,16,17], in addition to chronic pathological conditions such as nasal, cardiovascular, metabolic, renal, hepatic, and immuno-inflammatory diseases, as well as neurodegenerative and mental diseases such as depression [18,19,20,21,22,23]. The olfactory function is closely linked to the quality of life of all individuals, from the young to the elderly. In fact, people who suffer from olfactory dysfunction complain of food dissatisfaction and eating disorders, report a greater number of domestic accidents and, therefore, a reduced ability to protect themselves from dangers, and describe a negative impact on their emotional and mental health with consequent social isolation and the possible onset of depressive states [14,24,25,26]. This could explain why the elderly often show poor eating habits with the risk of developing conditions of malnutrition and/or increased body weight, social isolation, and the development of depressive states, as well as a greater possibility of incurring environmental hazards [24,27,28].

An active lifestyle in terms of both physical and cognitive activity is known to improve health conditions affected by a physiological decline associated with age. In particular, the risk factors of chronic pathologies associated with olfactory dysfunctions such as diabetes, obesity, cardiovascular, metabolic, mental, neo-degenerative and inflammatory diseases appear to be reduced by regular physical and/or mental exercise [21,26,29,30,31,32,33,34,35]. In this regard, a recent study conducted in our laboratories on an elderly population has highlighted a direct correlation between olfactory function and time spent in physical and cognitive activities; the most active individuals obtained significantly higher olfactory scores than the weakly active ones [36].

Lifestyle, metabolic state, state of hunger or satiety, sex, age, genetic predisposition, and state of health are all factors that affect the olfactory function of individuals. Most studies have focused on the effects of these parameters taken individually, so the first objective of this study was to assess the effect of body weight and/or sex on the olfactory function of elderly subjects (ES) who were classified as active or non-active in relation to their living habits. In fact, while the progressive sensory deterioration linked to age is commonly accepted, the presence of differences between individuals in their olfactory sensitivity that are sex-related is still a matter of debate [4,37,38,39]. Given the relationship between olfactory function and eating behavior, which, in turn, influences BMI and body weight, the second aim of this study was to estimate the effect of the olfactory function on BMI in elderly individuals based on their lifestyle (active or non-active) and sex (females or males). Furthermore, we looked for a link between each subject’s BMI and his/her TDI olfactory score and the time devoted (h/week) to life activities, both across populations and sexes.

## 2. Materials and Methods

### 2.1. Subjects

Senior volunteers (*n* = 133) who participated in this study were recruited in the metropolitan area of Cagliari and in the province of South Sardinia (Italy) and were classified as active Elderly Subjects (*n* = 65; 29 men, 36 women; age 67.7 ± 1.06 years) or non-active Elderly Subjects (*n* = 68; 32 men, 36 women, aged 70.5 ± 1.08 years), as reported in Sollai and Crnjar [36]. Healthy females and males aged >55 years who reported having a normal sense of smell were included in the panel, while those who reported the presence of chronic diseases (e.g., diabetes, neurodegeneration, severe cardiovascular disease, etc.) and/or acute diseases of their respiratory tract, were excluded. In addition, we excluded from the study individuals with a history of COVID-19 infection of less than 9 months. For each participant, a wall-mounted stadiometer (SECA) was used to measure height, expressed in cm, while a calibrated scale (TANITA) was used to evaluate the body weight expressed in Kg. The body mass index (BMI), determined through the ratio of weight to the square of height (kg/m^2^), was used to classify the weight condition of the subjects. Anthropometric and olfactory characteristics, lifestyle of the participants, and the number of elderly subjects with co-morbidities are reported in Table 1.

### 2.2. Assessment of Physical, Social and Cognitive Activity

Assessment of physical, social, and cognitive activities was performed by means of face-to-face interviews, as reported in Sollai and Crnjar [36]. Briefly, subjects were asked to answer questions related to the number of hours per day and number of days per week they spent on: (a) walking or running for exercise, heavy housework or gardening, field work, swimming, or dancing; (b) meetings, planning and/or attending events, and attending lectures; (c) reading a book and/or solving puzzles. The total number of hours devoted to each of these activities was added together and reported as hours of weekly activity (hours/week). According to Buchman et al. [29], we classified the motor activity as “exercise-physical activity” (E-PA) and as “non exercise-physical activity” (nE-PA) as the hours dedicated to social and cognitive activities.

### 2.3. Olfactory Sensitivity Sssessment

The “Sniffin’ Sticks” battery test [40] was used to assess each subject’s orthonasal olfactory function, which consists of three subtests for olfactory threshold (T-test), odor discrimination (D-test), and odor identification (I-test). For the T-test, the researcher has a kit of 48 pens arranged in 16 triplets; each triplet has two pens containing a solvent and a third pen (target pen) loaded with n-butanol solution at escalating concentrations. A scale reversal begins if the subject correctly identifies the target pen twice in a row. When the seventh scale reversal is accomplished, the test is finished, and the T-test score is calculated using the average of the last four reversals. For the D-test, the researcher has 16 triplets, each consisting of two identical pens and one loaded with a distinct odor (target pen). The aim for the participant is to find the target pen. From 0 to 16, the D-test score correlates with the number of correct answers. For the I-test, individuals have to sniff 16 pens containing odors they are familiar with. In a forced-choice approach, the participant must choose one of four items each time he/she smells a pen. From 0 to 16, the score is given by the sum of correct answers.

The sum of the T-test, D-test, and I-test values is used to obtain the total TDI olfactory score. By using the age and gender normalized values reported in Hummel et al. [41], each individual was classified as normosmic or hyposmic.

### 2.4. Data Analyses

One-way ANOVA was used to test for a significant difference between the two populations (active ES vs. non-active ES) in their BMI.

Factorial ANOVA was used to test for a significant interaction between: (a) population (active or non-active) × BMI status (normal weight or overweight), population × sex (female or male) and population × sex × BMI status on the TDI score; (b) population × TDI olfactory status (normosmic or hyposmic), population × sex and population × sex × TDI olfactory status on the BMI.

Fisher’s LSD test (*p* values < 0.05 were judged significant) was used for post-analysis. STATISTICA for WINDOWS (version 7.0; StatSoft Inc., Tulsa, OK, USA) was used for the statistical analysis.

The relationships between BMI and TDI olfactory score, BMI, and hours/week of exercise or non-exercise physical activities, for each population and sex separately, was evaluated by means of the Pearson’s correlation coefficient. GraphPad Prism 6 (GraphPad Software, San Diego, CA, USA) was used for the statistical analysis. *p* Values < 0.05 were significant.

## 3. Results

### 3.1. Olfactory Scores and BMI in Female and Male Actine or Non-Active Elderly Subjects

**Figure 1:**
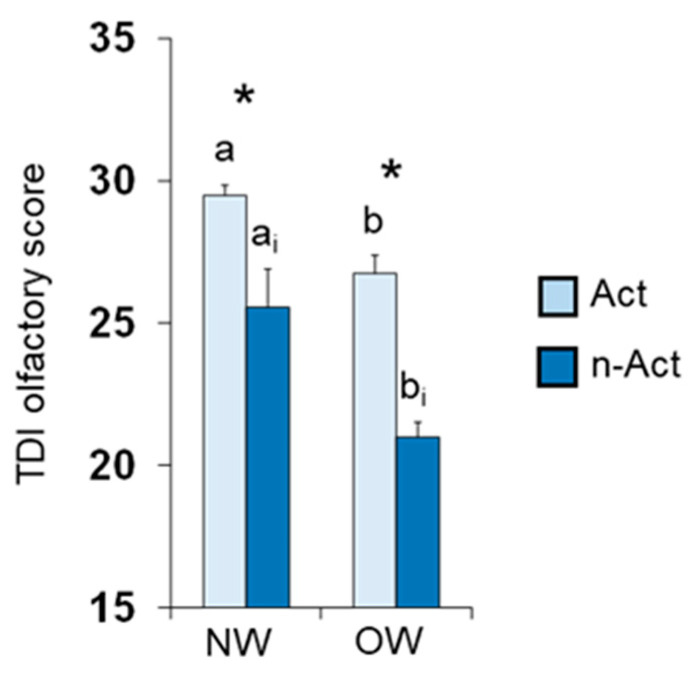
Mean value (±SEM) of the TDI olfactory score obtained by active ES (*n* = 65) and non-active ES (*n* = 68) classified as Normal Weight (NW; active *n* = 49, non-active *n* = 15) or Overweight (OW; active *n* = 16, non-active *n* = 53) according to their BMI value. Different letters indicate significant differences between normal weight and overweight active ES (a–b) or non-active ES (a_i_–b_i_) (*p* < 0.0005; Fisher’s LSD test subsequent to two-way ANOVA). Asterisk indicates significant differences between active and non-active ES normal weight (*p* = 0.011; Fisher’s LSD test subsequent to two-way ANOVA) or overweight (*p* < 0.0001; Fisher’s LSD test subsequent to two-way ANOVA).

Figure 1 shows mean values ± SEM of the TDI olfactory score obtained by the active Elderly Subjects and non-active Elderly Subjects according to their BMI status (normal weight, NW or overweight, OW). Post hoc comparisons subsequent to two-way ANOVA revealed that, for both active ES and non-active ES, normal weight individuals achieved olfactory scores that were significantly higher than those obtained for overweight ones (active ES: *p* < 0.0005; non-active ES: *p* < 0.0001; Fisher’s LSD test). Furthermore, in both normal weight and overweight individuals, active ES obtained TDI olfactory scores higher than non-active ES (NW: *p* = 0.011; OW: *p* < 0.0001; Fisher’s LSD test).

**Figure 2:**
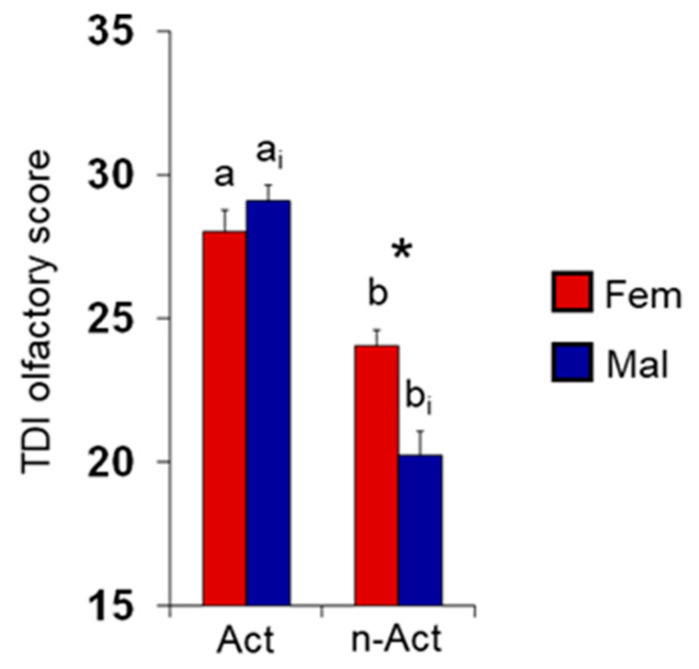
Mean value (±SEM) of the TDI olfactory score obtained by active ES (*n* = 65) and non-active ES (*n* = 68) considered separately for females (active *n* = 36, non-active *n* = 36) and males (active *n* = 29, non-active *n* = 32). Different letters indicate significant differences between active and non-active females (a–b) or males (a_i_–b_i_) (*p* < 0.0001; Fisher’s LSD test subsequent to two-way ANOVA). Asterisk indicates significant differences between female and male non-active ES (*p* < 0.0005; Fisher’s LSD test subsequent to two-way ANOVA).

The mean ± SEM value of the TDI olfactory score obtained by active ES and non-active ES according to their sex (females or males) are shown in Figure 2. In detail, two-way ANOVA revealed a significant interaction between population × sex on the TDI olfactory score (F 1,129 = 12,894; *p* < 0.0005) and post hoc comparisons showed that both female and male active ES reached TDI scores higher than female and male non-active ES (*p* < 0.0001; Fisher’s LSD test). Among non-active ES, females achieved TDI score higher than males (*p* = 0.0001; Fisher’s LSD test), while no difference was found between sexes among active ES (*p* > 0.05).

**Figure 3:**
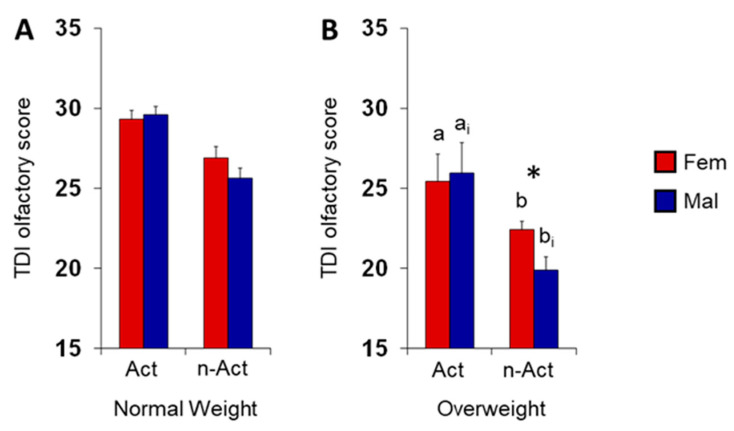
Mean value (±SEM) of the TDI olfactory score obtained by females (Fem) and males (Mal) ES according to their lifestyle (active or non-active) and BMI status ((**A**) normal weight or (**B**) overweight). Active ES: F NW *n* = 24, F OW *n* = 12, M NW *n* = 25, active M OW *n* = 4. Non-active ES: F NW *n* = 13, F OW *n* = 23, M NW *n* = 2, M OW *n* = 30. Different letters indicate significant differences between both active and non-active females (a–b; p = 0.019) and males (a_i_–b_i_; *p* = 0.0017). Asterisk indicates significant differences between females and males non-active ES (*p* = 0.011; Fisher’s LSD test subsequent to three-way ANOVA).

Figure 3A,B shows mean values ± SEM of the TDI olfactory score obtained by females and males according to their lifestyle (active or non-active) and BMI status (normal-weight or overweight). For overweight individuals, post hoc comparisons subsequent to three-way ANOVA (F 1,125 = 1.14; *p* = 0.29) revealed that the TDI olfactory scores were higher in both females and male active ES than in females and male non-active ES (*p* = 0.001); in addition, a significant difference was found between sexes, with females performing better than males (*p* = 0.011). Instead, no difference was found in the TDI scores between lifestyles (active or non-active) and sexes (female or male) for normal weight individuals (*p* > 0.05).

**Figure 4:**
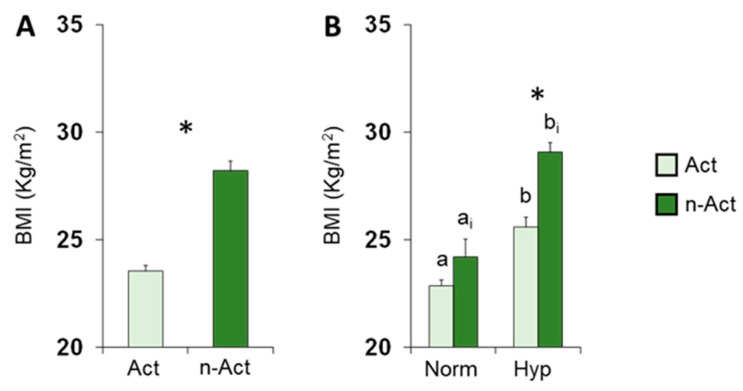
Mean value (±SEM) of BMI determined in active (*n* = 65) and non-active (*n* = 68) ES (**A**) and according to their TDI olfactory status (Nor = normosmia; Hyp = hyposmia) (**B**). Active ES: Nor *n* = 49, Hyp *n* = 16. Non-active ES: Nor *n* = 12, Hyp *n* = 56. (**A**) Asterisk indicates significant differences between active and non-active ES (*p* < 0.0001; Fisher’s LSD test subsequent to one-way ANOVA). (**B**) Different letters indicate significant differences between normosmic and hyposmic individuals among active (a–b) or non-active (a_i_–b_i_) ES (*p* < 0.001; Fisher’s LSD test subsequent to two-way ANOVA). Asterisk indicates significant differences between active and non-active ES within the same olfactory status (*p* < 0.0001; Fisher’s LSD test subsequent to two-way ANOVA).

The mean values ± SEM of BMI determined in active ES and non-active ES are shown in Figure 4A. One-way ANOVA revealed that BMI of active ES was significantly lower than that of non-active ES (F 1,131 = 75.45; *p* < 0.0001). Figure 3B shows the same data according to their TDI olfactory status. Post hoc comparisons subsequent to two-way ANOVA (F 1,129 = 3.44, *p* = 0.066) highlighted that hyposmic individuals showed higher BMI values than normosmic ones (active ES, *p* = 0.0005; non-active ES, *p* < 0.0001; Fisher’s LSD test) and that hyposmic non-active ES had a higher BMI than hyposmic active ES (*p* < 0.0001; Fisher’s LSD test). Instead, no difference was observed between normosmic active and non-active ES (*p* > 0.05; Fisher’s LSD test).

**Figure 5:**
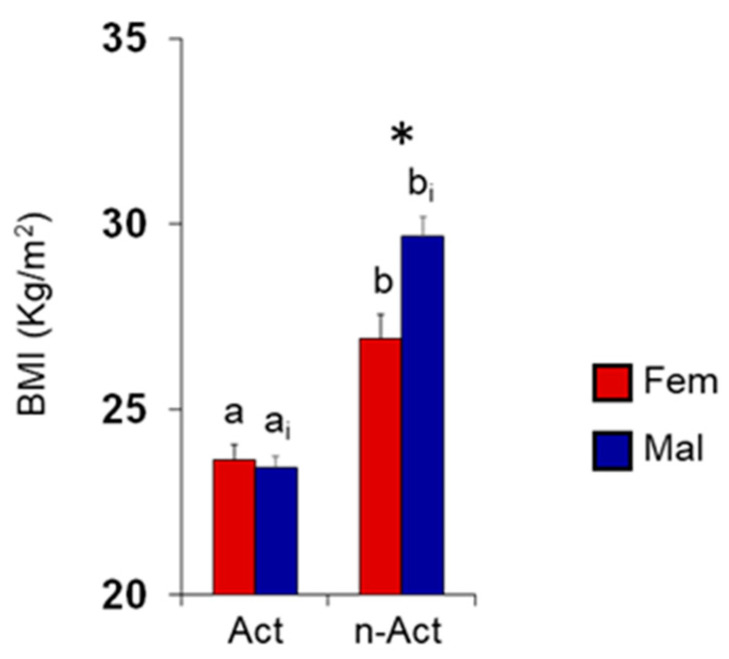
Mean value (± SEM) of BMI determined in females (F) and males (M) ES according to their lifestyle (active or non-active). Active ES: F *n* = 36, M *n* = 29. Non-active ES: F *n* = 36, M *n* = 321. Different letters indicate significant differences between active and non-active ES among females (a–b) or males (a_i_–b_i_) (*p* < 0.0001; Fisher’s LSD test subsequent to two-way ANOVA). Asterisk indicates significant differences between sexes among the same population (*p* = 0.0002; Fisher’s LSD test subsequent to two-way ANOVA).

Figure 5 shows mean values ± SEM of the BMI obtained by females and males according to their lifestyle (active or non-active). Two-way ANOVA revealed significant interactions of lifestyle x sex on the BMI (F 1,129 = 8.39, *p* = 0.005); post hoc comparisons showed that non-active females and males had a higher BMI than the active ones (*p* < 0.0001; Fisher’s LSD test) and that non-active males had a higher BMI than non-active females (*p* = 0.0002; Fisher’s LSD test). Instead, no difference was observed between active female and male ES (*p* > 0.05; Fisher’s LSD test).

**Figure 6:**
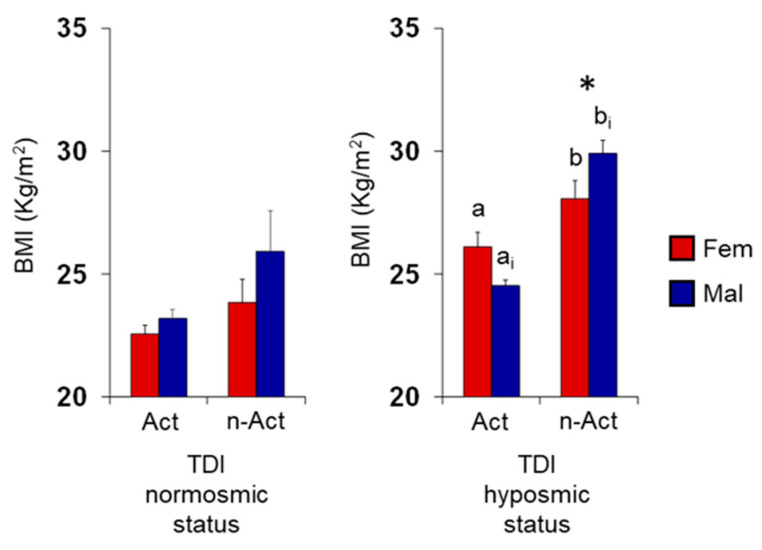
Mean (±SE) values of the BMI determined determined in active and non-active ES according to their TDI olfactory status (Nor = normosmia; Hyp = hyposmia) and sex. Active ES: F Nor *n* = 25, F Hyp *n* = 11, M Nor *n* = 24, M Hyp *n* = 5. Non-active ES: F Nor *n* = 10, F Hyp *n* = 26, M Nor *n* = 2, M Hyp *n* = 30. Different letters indicate significant differences between active and non-active ES among females (a–b) or males (a_i_–b_i_) (*p* ≤ 0.038; Fisher’s LSD test subsequent to three-way ANOVA). Asterisk indicates significant differences between sexes among the same population (*p* < 0.001; Fisher’s LSD test subsequent to three-way ANOVA).

Figure 6 represents mean values ± SEM of the BMI obtained by females and males according to their lifestyle (active or non-active) and TDI olfactory status (normosmia or hyposmia). Post hoc comparisons subsequent to three-way ANOVA (F 1,125 = 0.54, *p* = 0.47) highlighted that non-active hyposmic individuals showed BMI higher than active ones (*p* ≤ 0.038; Fisher’s LSD test) and that male non-active ES had a higher BMI than female non-active ES (*p* < 0.001; Fisher’s LSD test). Instead, for normosmic individuals, no difference was observed between lifestyles or sexes (*p* > 0.05; Fisher’s LSD test).

### 3.2. Correlation Analysis

**Figure 7:**
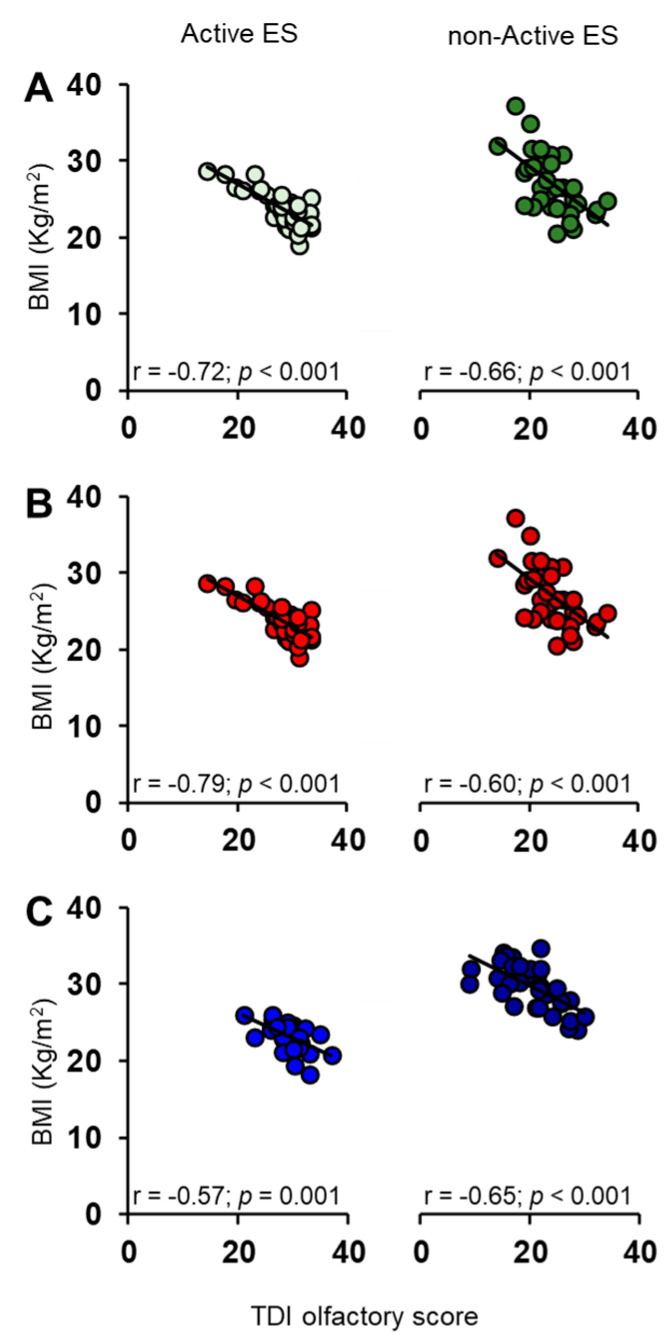
Correlation analysis between BMI and TDI olfactory score obtained by each active and non-active ES, considering them all together (**A**) and separately in females (**B**) and males (**C**).

Pearson’s correlation test was used to verify for a correlation between BMI and TDI olfactory scores in active and non-active ES (Figure 7). The results show that the TDI olfactory score obtained by each subject and his/her BMI are negatively correlated, both when considered all together or for females and males separately.

**Figure 8:**
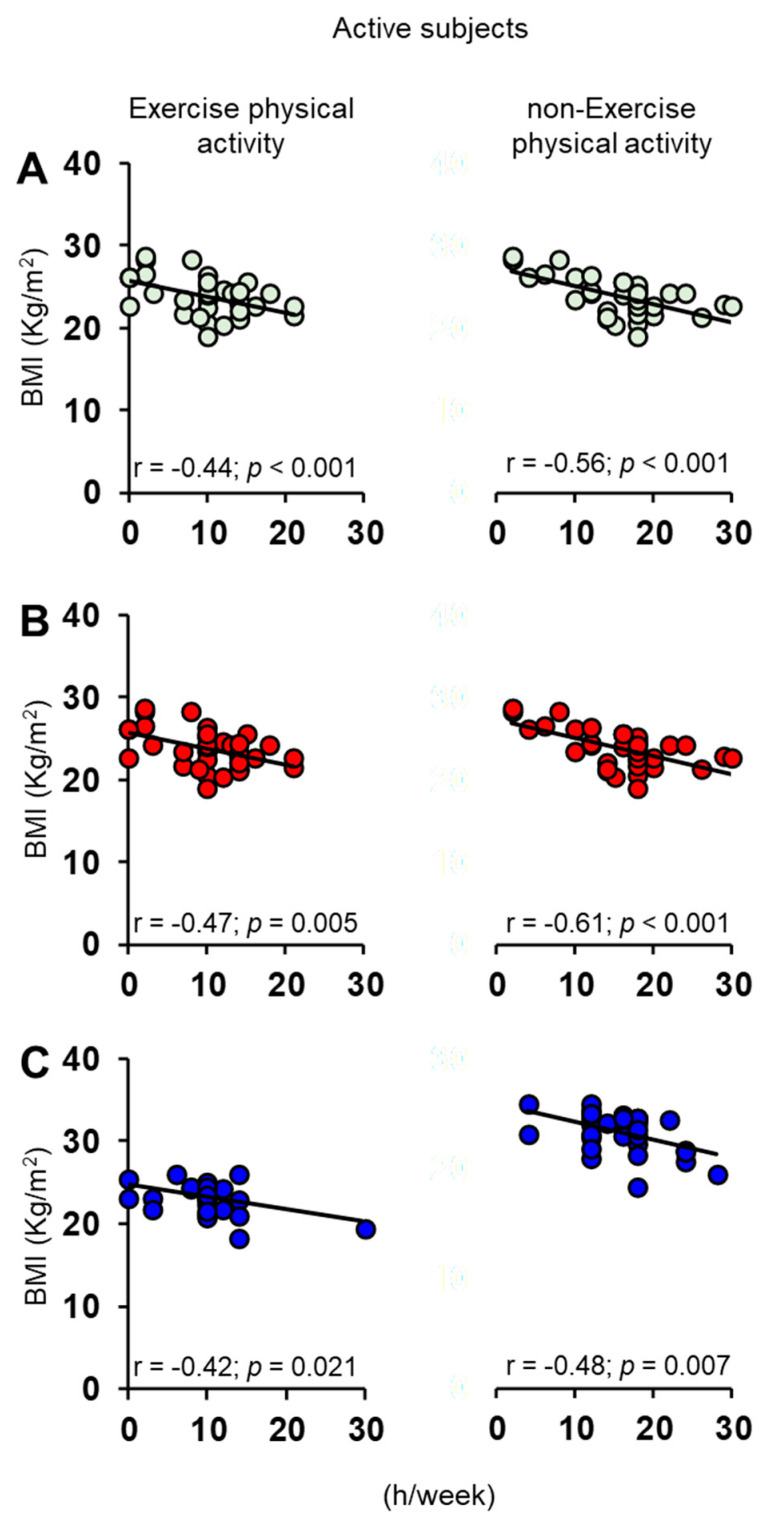
Correlation analysis between BMI and the number of weekly hours (h/week) that each active subject dedicated to exercise and non-exercise physical activities, considering them all together (**A**) and separately in females (**B**) and males (**C**).

**Figure 9:**
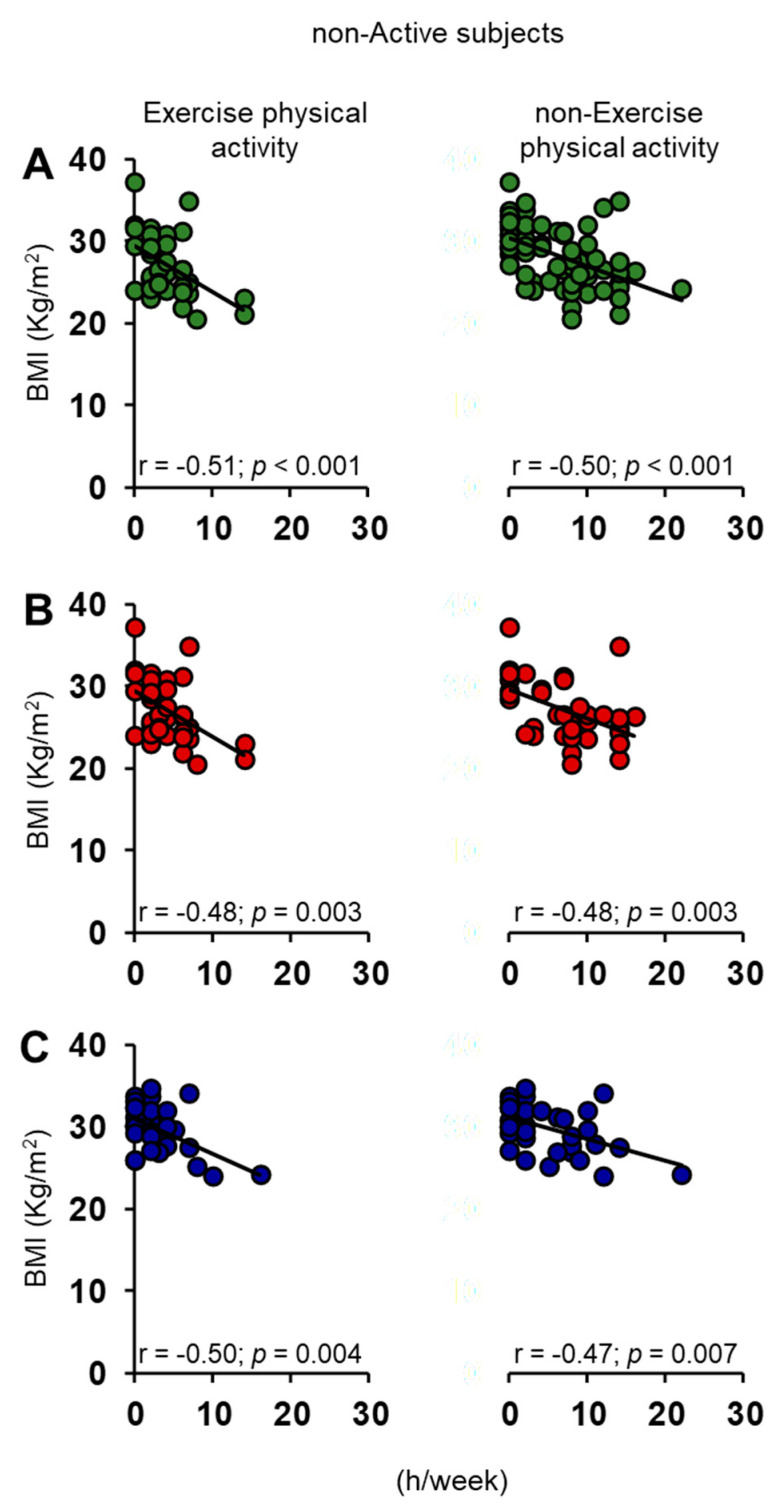
Correlation analysis between BMI and the number of weekly hours (h/week) that each non-active subject dedicated to exercise and non-exercise physical activities, considering them all together (**A**) and separately in females (**B**) and males (**C**).

The Pearson’s correlation test was also used to check for a relationship between BMI of each individual with his/her exercise and non-exercise physical activity (h/week). For both active (Figure 8) and non-active (Figure 9) ES, we found a negative correlation both when individuals were considered all together and when they were divided into females and males.

## 4. Discussion

We have previously shown that the elderly individuals with an active lifestyle present a better olfactory function than those with a non-active lifestyle and that the number of hours spent in both exercise and non-exercise physical activities is directly correlated with the olfactory score obtained by the individuals of both populations (active or non-active) [36]. One of the main functions of the olfactory system is to influence food choices and food intake, participating in the modulation of a meal size and contributing to determining body weight and BMI [42,43,44,45,46]. People with impaired olfactory function sometimes report having changed their eating habits, seeking more appetizing foods, but perceiving them as less tasty and less pleasant [28,47,48].

On this basis, the first purpose of this study was to evaluate the effect of body weight and sex on the olfactory function of active and non-active elderly subjects (ES). The results we obtained indicate that both normal and overweight active ES of both females and males achieve significantly higher TDI olfactory scores than non-active ones. Normal weight individuals achieve higher TDI olfactory scores than overweight individuals, regardless of their active or non-active lifestyle; on the other hand, as regards sex, non-active females obtain higher TDI scores than non-active males, while no difference emerged between females and males for active ES. In general, our results show that among normal weight individuals, the TDI olfactory score does not significantly change between active and non-active ES and between females and males. In contrast, among overweight individuals, both non-active females and males obtain lower TDI scores than active ones; moreover, when we consider only non-active ES, males score significantly lower than females. These findings suggest that the condition of normal weight is inversely associated with the olfactory deficit related to the non-active versus active lifestyle. This could be due to the fact that olfactory function is modulated by the circulating levels of peptides that regulate energy metabolism: orexigenic peptides such as ghrelin increase olfactory sensitivity, while anorexigenic ones such as leptin and insulin decrease it [43,49,50,51,52,53]. We suggest that the reduced olfactory acuity associated with weight gain is the result of the opposite effect of increased leptin levels, with an inhibitory action on the mitral cells of the olfactory bulb [51,52,53] and a decrease in circulating levels of ghrelin, with consequent reduction of its stimulating effect on the olfactory function [54]. These results are in agreement with previous studies reporting a reduction in olfactory function related to an increase in body weight [46,55,56,57].

The second aim of this study was to evaluate the role of smell on body weight by measuring the BMI in relation to the different lifestyle and sex of the ES. We found that both active and non-active ES classified as normosmic have a significantly lower BMI than those classified as hyposmic and, among hyposmic individuals, the non-active ES exhibit a higher BMI than active ones. Furthermore, both females and males classified as active ES exhibit lower BMI than those classified as non-active and, among non-active individuals, females exhibit a lower BMI than males. In general, it emerges that a normosmia condition is inversely related to an increase in body weight linked to a non-active lifestyle, both in females and males. On the other hand, among hyposmic ES, the differences in weight gain become significant in relation to the active or non-active lifestyle and also between females and males, at least as regards the non-active ES. On the basis of these findings, we could speculate that lifestyle may mask possible sex-related differences and that a normal olfactory function may balance not only sex-related differences but also those due to different lifestyles. In fact, the differences related to lifestyle and sex are observed only among those ES exhibiting an olfactory dysfunction, particularly in the case of a non-active lifestyle.

The last objective of this study was to verify the relationship between the BMI and TDI olfactory score obtained by each elderly subject of both groups and between the BMI and the number of weekly hours dedicated to exercise and non-exercise physical activities by considering both females and males separately and jointly. The negative relationships we found between BMI and TDI olfactory score and between BMI and exercise/non-exercise physical activities support the results shown above and suggest that lifestyle, smell, and BMI are linked in two ways. As we have previously shown [36], elderly individuals who report having a non-stimulating lifestyle in terms of physical, social, and cognitive activity show a reduction in their olfactory function. Since olfaction influences eating behavior and the responses of the cephalic phase of food intake, that is, in the processes of the beginning and the end of the meal [58], this leads hyposmic individuals not only to prefer sweet and fat-rich foods instead of fruits and vegetables but also to add condiments and spices to compensate for the reduced gratification due to their reduced olfactory function [28,47,59,60]. This also prolongs the intake of these foods due to a delay in reaching satiety [61,62], which results in a body weight increase in these individuals who, as our data show, have a higher BMI than those with a normal olfactory function.

In turn, a high BMI impacts the olfactory function of individuals by affecting their lifestyle by reducing their exercise activity due to a higher body weight that limits movements but also social activities. As already mentioned, the increase in body weight is associated with metabolic changes and circulating hormone levels that influence the olfactory function which, on the other hand, influences the sociability of individuals leading the elderly to reduce their social relationships because of their inability to enjoy the pleasures of food (e.g., in situations of conviviality, such as a dinner with friends or a party with relatives) and/or due to a state of insecurity due to one’s body odor [27,63,64].

In elderly subjects, these relationships are of particular importance because olfactory dysfunction, weight gain, and lifestyle are also associated with other pathologies, such as hypertension, diabetes, depression, metabolic disorders, obesity, and inflammatory diseases, which co-act to cause brain dysfunction and neurodegeneration [21,26,29,30,31,32,33,34,57,65,66,67]. Therefore, understanding the mechanisms and factors involved and their interactions can be useful for improving the health status of individuals such as the elderly, who often struggle with the behavioral and cognitive limitations that characterize their age. We could speculate that a normal condition in one of the factors involved (lifestyle, olfaction, or body weight) may compensate the negative aspects related to the other. The normal weight condition is inversely related to the olfactory dysfunction linked to lifestyle and sex differences, while the normosmia condition is inversely associated with the increase due to lifestyle and sex-related differences.

## 5. Conclusions

In conclusion, given the close relationship between olfactory function, BMI, and exercise and non-exercise physical activity, it can be assumed that ES may benefit from an active lifestyle. Indeed, an active lifestyle associated with a normal olfactory function, which plays an important role in individuals’ eating behavior, can have a positive effect on their body weight and BMI, and that this benefit appears to be the same for both women and men. In particular, the fact that the relationship between BMI and non-exercise physical activity is comparable to that between BMI and exercise physical activity may be of relevant importance for ES with limited mobility. This means that elderly people with reduced mobility can obtain benefits for their olfactory function and for their body weight from an active lifestyle, even if only in cognitive and social terms.

## Figures and Tables

**Table 1:** Anthropometric and olfactory characteristics, lifestyle of the participants, and number of elderly subjects with co-morbidities.

Panel	Active ES	n-Active ES
*n*	65	68
Age (years)	67.7 ± 1.06	70.5 ± 1.08
BMI (Kg/m^2^)	23.6 ± 0.27	28.2 ± 0.46
TDI olfactory score	28.5 ± 0.48	22.3 ± 0.53
Exercise activities	10.1 ± 0.67	3.56 ± 0.42
non-Exercise activities	15.9 ± 0.75	6.46 ± 0.65
ES with co-morbidities		
Hypertension	6	5
Hyperglycemia	4	3
Hypercholesterolemia	2	2
Rheumatoid arthritis	1	1

## Data Availability

The data presented in this study are available on request from the corresponding author. The data are not publicly available due to restrictions (e.g., privacy or ethical).

## References

[1] Calderón-Garcidueñas L., Franco-Lira M., Henríquez-Roldán C., Osnaya N., González-Maciel A., Reynoso-Robles R., Villarreal-Calderon R., Herritt L., Brooks D., Keefe S. (2010). Urban air pollution: Influences on olfactory function and pathology in exposed children and young adults. Exp. Toxicol. Pathol..

[2] Crnjar R., Solari P., Sollai G. (2023). The Human Nose as a Chemical Sensor in the Perception of Coffee Aroma: Individual Variability. Chemosensors.

[3] Keller A., Zhuang H., Chi Q., Vosshall L.B., Matsunami H. (2007). Genetic variation in a human odorant receptor alters odour perception. Nature.

[4] Melis M., Tomassini Barbarossa I., Crnjar R., Sollai G. (2022). Olfactory Sensitivity Is Associated with Body Mass Index and Polymorphism in the Voltage-Gated Potassium Channels Kv1.3. Nutrients.

[5] Melis M., Tomassini Barbarossa I., Hummel T., Crnjar R., Sollai G. (2021). Effect of the rs2890498 polymorphism of the OBPIIa gene on the human ability to smell single molecules. Behav. Brain Res..

[6] Menashe I., Abaffy T., Hasin Y., Goshen S., Yahalom V., Luetje C.W., Lancet D. (2007). Genetic elucidation of human hyperosmia to isovaleric acid. PLoS Biol..

[7] Sollai G., Melis M., Magri S., Usai P., Hummel T., Tomassini Barbarossa I., Crnjar R. (2019). Association between the rs2590498 polymorphism of Odorant Binding Protein (OBPIIa) gene and olfactory performance in healthy subjects. Behav. Brain Res..

[8] Sollai G., Melis M., Tomassini Barbarossa I., Crnjar R. (2022). A polymorphism in the human gene encoding OBPIIa affects the perceived intensity of smelled odors. Behav. Brain Res..

[9] Sollai G., Tomassini Barbarossa I., Usai P., Hummel T., Crnjar R. (2020). Association between human olfactory performance and ability to detect single compounds in complex chemical mixtures. Physiol. Behav..

[10] Sorokowska A., Sorokowski P., Frackowiak T. (2015). Determinants of human olfactory performance: A cross-cultural study. Sci. Total Environ..

[11] Sorokowska A., Sorokowski P., Hummel T., Huanca T. (2013). Olfaction and environment: Tsimane’ of Bolivian rainforest have lower threshold of odor detection than industrialized German people. PLoS ONE.

[12] Cain W.S., Stevens J.C. (1989). Uniformity of olfactory loss in aging. Ann. N. Y. Acad. Sci..

[13] Doty R.L., Shaman P., Applebaum S.L., Giberson R., Siksorski L., Rosenberg L. (1984). Smell identification ability: Changes with age. Science.

[14] Min H.J., Kim S.M., Han D.H., Kim K.S. (2021). The sniffing bead system, an olfactory dysfunction screening tool for geriatric subjects: A cross-sectional study. BMC Geriatr..

[15] Attems J., Walker L., Jellinger K.A. (2015). Olfaction and Aging: A Mini-Review. Gerontology.

[16] Doty R.L., Kamath V. (2014). The influences of age on olfaction: A review. Front. Psychol..

[17] Seiberling K.A., Conley D.B. (2004). Aging and olfactory and taste function. Otolaryngol. Clin. N. Am..

[18] Boesveldt S., Verbaan D., Knol D.L., Visser M., van Rooden S.M., van Hilten J.J., Berendse H.W. (2008). A comparative study of odor identification and odor discrimination deficits in Parkinson’s disease. Movement Disord..

[19] Larsson M., Semb H., Winblad B., Amberla K., Wahlund L.O., Bäckman L. (1999). Odor identification in normal aging and early Alzheimer’s disease: Effects of retrieval support. Neuropsychology.

[20] Makizako M., Makizako H., Doi T., Uemura K., Tsutsumimoto K., Miyaguchi H., Shimada H. (2014). Olfactory identification and cognitive performance in community-dwelling older adults with mild cognitive impairment. Chem. Senses.

[21] Perricone C., Shoenfeld N., Agmon-Levin N., de Carolis C., Perricone R., Shoenfeld Y. (2013). Smell and autoimmunity: A comprehensive review. Clin. Rev. Allergy Immunol..

[22] Steinbach S., Reindl W., Dempfle A., Schuster A., Wolf P., Hundt W., Huber W. (2013). Smell and taste in inflammatory bowel disease. PLoS ONE.

[23] Velluzzi F., Deledda A., Lombardo M., Fosci M., Crnjar R., Grossi E., Sollai G. (2023). Application of Artificial Neural Networks (ANN) to Elucidate the Connections among Smell, Obesity with Related Metabolic Alterations, and Eating Habit in Patients with Weight Excess. Metabolites.

[24] Croy I., Nordin S., Hummel T. (2014). Olfactory Disorders and Quality of Life—An Updated Review. Chem. Senses.

[25] Hummel T., Nordin S. (2005). Olfactory disorders and their consequences for quality of life. Acta Oto-Laryngol..

[26] Pinto J.M., Wroblewski K.E., Kern D.W., Schumm L.P., McClintock M.K. (2014). Olfactory dysfunction predicts 5-year mortality in older adults. PLoS ONE.

[27] Boesveldt S., Parma V. (2021). The importance of the olfactory system in human well-being, through nutrition and social behavior. Cell Tissue Res..

[28] Duffy V.B., Backstrand J.R., Ferris A.M. (1995). Olfactory dysfunction and related nutritional risk in free-living, elderly women. J. Am. Diet. Assoc..

[29] Buchman A.S., Boyle P.A., Yu L., Shah R.C., Wilson R.S., Bennett D.A. (2012). Total daily physical activity and the risk of AD and cognitive decline in older adults. Neurology.

[30] Cotman C.W., Berchtold N.C., Christie L.A. (2007). Exercise builds brain health: Key roles of growth factor cascades and inflammation. Trends Neurosci..

[31] Croy I., Symmank A., Schellong J., Hummel C., Gerber J., Joraschky P., Hummel T. (2014). Olfaction as a marker for depression in humans. J. Affect. Disord..

[32] Franco O., De Laet C., Peeters A., Jonker J., Mackenbach J., Nusselder W. (2005). Effects of Physical Activity on Life Expectancy With Cardiovascular Disease. Arch. Intern. Med..

[33] Kim S.J., Windon M.J., Lin S.Y. (2019). The association between diabetes and olfactory impairment in adults: A systematic review and meta-analysis. Laryngoscope Investig. Otolaryngol..

[34] Ross G.W., Petrovitch H., Abbott R.D., Tanner C.M., Popper J., Masaki K., Launer L., White L.R. (2008). Association of olfactory dysfunction with risk for future Parkinson’s disease. Ann. Neurol..

[35] Wilson R.S., Schneider J.A., Arnold S.E., Tang Y., Boyle P.A., Bennett D.A. (2007). Olfactory Identification and Incidence of Mild Cognitive Impairment in Older Age. Arch. Gen. Psychiatry.

[36] Sollai G., Crnjar R. (2021). Age-Related Olfactory Decline Is Associated With Levels of Exercise and Non-exercise Physical Activities. Front. Aging Neurosci..

[37] Kern D.W., Wroblewski K.E., Schumm L.P., Pinto J.M., Chen R.C., McClintock M.K. (2014). Olfactory Function in Wave 2 of the National Social Life, Health, and Aging Project. J. Gerontol. Ser. B.

[38] Sorokowska A., Schriever V.A., Gudziol V., Hummel C., Hähner A., Iannilli E., Sinding C., Aziz M., Seo H.S., Negoias S. (2015). Changes of olfactory abilities in relation to age: Odor identification in more than 1400 people aged 4 to 80 years. Eur. Arch. Oto-Rhino-Laryngol..

[39] Sorokowski P., Karwowski M., Misiak M., Marczak M.K., Dziekan M., Hummel T., Sorokowska A. (2019). Sex Differences in Human Olfaction: A Meta-Analysis. Front. Psychol..

[40] Hummel T., Sekinger B., Wolf S.R., Pauli E., Kobal G. (1997). ‘Sniffin’ sticks’: Olfactory performance assessed by the combined testing of odor identification, odor discrimination and olfactory threshold. Chem. Senses.

[41] Hummel T., Kobal G., Gudziol H., Mackay-Sim A. (2007). Normative data for the “Sniffin’ Sticks” including tests of odor identification, odor discrimination, and olfactory thresholds: An upgrade based on a group of more than 3000 subjects. Eur. Arch. Otorhinolaryngol..

[42] Besser G., Erlacher B., Aydinkoc-Tuzcu K., Liu D.T., Pablik E., Niebauer V., Koenighofer M., Renner B., Mueller C.A. (2020). Body-Mass-Index Associated Differences in Ortho- and Retronasal Olfactory Function and the Individual Significance of Olfaction in Health and Disease. J. Clin. Med..

[43] Palouzier-Paulignan B., Lacroix M.C., Aimé P., Baly C., Caillol M., Congar P., Julliard A.K., Tucker K., Fadool D.A. (2012). Olfaction under metabolic influences. Chem. Senses.

[44] Stafford L.D., Whittle A. (2015). Obese individuals have higher preference and sensitivity to odor of chocolate. Chem. Senses.

[45] Stevenson R.J. (2010). An initial evaluation of the functions of human olfaction. Chem. Senses.

[46] Velluzzi F., Deledda A., Onida M., Loviselli A., Crnjar R., Sollai G. (2022). Relationship between Olfactory Function and BMI in Normal Weight Healthy Subjects and Patients with Overweight or Obesity. Nutrients.

[47] Manesse C., Ferdenzi C., Sabri M., Bessy M., Rouby C., Faure F., Bellil D., Jomain S., Landis B.N., Hugentobler M. (2017). Dysosmia-Associated Changes in Eating Behavior. Chemosens. Percept..

[48] Peng M., Coutts D., Wang T., Cakmak Y.O. (2019). Systematic review of olfactory shifts related to obesity. Obes. Rev..

[49] Gschwend O., Beroud J., Vincis R., Rodriguez I., Carleton A. (2016). Dense encoding of natural odorants by ensembles of sparsely activated neurons in the olfactory bulb. Sci. Rep..

[50] Julliard A.K., Al Koborssy D., Fadool D.A., Palouzier-Paulignan B. (2017). Nutrient Sensing: Another Chemosensitivity of the Olfactory System. Front. Physiol..

[51] Sirotin Y.B., Shusterman R., Rinberg D. (2015). Neural Coding of Perceived Odor Intensity. eNeuro.

[52] Sun C., Tang K., Wu J., Xu H., Zhang W., Cao T., Zhou Y., Yu T., Li A. (2019). Leptin modulates olfactory discrimination and neural activity in the olfactory bulb. Acta Physiol..

[53] Tschöp M., Weyer C., Tataranni P.A., Devanarayan V., Ravussin E., Heiman M.L. (2001). Circulating ghrelin levels are decreased in human obesity. Diabetes.

[54] Connor E.E., Zhou Y., Liu G.E. (2018). The essence of appetite: Does olfactory receptor variation play a role?. J. Anim. Sci..

[55] Fernández-Aranda F., Agüera Z., Fernández-García J.C., Garrido-Sanchez L., Alcaide-Torres J., Tinahones F.J., Giner-Bartolomé C., Baños R.M., Botella C., Cebolla A. (2016). Smell-taste dysfunctions in extreme weight/eating conditions: Analysis of hormonal and psychological interactions. Endocrine.

[56] Fernandez-Garcia J.C., Alcaide J., Santiago-Fernandez C., Roca-Rodriguez M.M., Aguera Z., Baños R., Botella C., de la Torre R., Fernandez-Real J.M., Fruhbeck G. (2017). An increase in visceral fat is associated with a decrease in the taste and olfactory capacity. PLoS ONE.

[57] Sollai G., Melis M., Mastinu M., Paduano D., Chicco F., Magri S., Usai P., Hummel T., Barbarossa I.T., Crnjar R. (2021). Olfactory Function in Patients with Inflammatory Bowel Disease (IBD) Is Associated with Their Body Mass Index and Polymorphism in the Odor Binding-Protein (OBPIIa) Gene. Nutrients.

[58] Power M.L., Schulkin J. (2008). Anticipatory physiological regulation in feeding biology: Cephalic phase responses. Appetite.

[59] Egecioglu E., Skibicka K.P., Hansson C., Alvarez-Crespo M., Friberg P.A., Jerlhag E., Engel J.A., Dickson S.L. (2011). Hedonic and incentive signals for body weight control. Rev. Endocr. Metab. Disord..

[60] Postma E., Graaf C., Boesveldt S. (2019). Food preferences and intake in a population of Dutch individuals with self-reported smell loss: An online survey. Food Qual. Prefer..

[61] Albrecht J., Schreder T., Kleemann A.M., Schöpf V., Kopietz R., Anzinger A., Demmel M., Linn J., Kettenmann B., Wiesmann M. (2009). Olfactory detection thresholds and pleasantness of a food-related and a non-food odour in hunger and satiety. Rhinology.

[62] Stafford L.D. (2016). Olfactory Specific Satiety depends on degree of association between odour and food. Appetite.

[63] Boesveldt S., Postma E.M., Boak D., Welge-Luessen A., Schöpf V., Mainland J.D., Martens J., Ngai J., Duffy V.B. (2017). Anosmia-A Clinical Review. Chem. Senses.

[64] Temmel A.F., Quint C., Schickinger-Fischer B., Klimek L., Stoller E., Hummel T. (2002). Characteristics of olfactory disorders in relation to major causes of olfactory loss. Arch. Otolaryngol. Head Neck Surg..

[65] Graves A.B., Bowen J.D., Rajaram L., McCormick W.C., McCurry S.M., Schellenberg G.D., Larson E.B. (1999). Impaired olfaction as a marker for cognitive decline: Interaction with apolipoprotein E epsilon4 status. Neurology.

[66] Hamer M., Sabia S., Batty G.D., Shipley M.J., Tabák A.G., Singh-Manoux A., Kivimaki M. (2012). Physical activity and inflammatory markers over 10 years: Follow-up in men and women from the Whitehall II cohort study. Circulation.

[67] Wilson R.S., Arnold S.E., Schneider J.A., Boyle P.A., Buchman A.S., Bennett D.A. (2009). Olfactory impairment in presymptomatic Alzheimer’s disease. Ann. N. Y. Acad. Sci..

